# Key quality parameter comparison of mesenchymal stem cell product cryopreserved in different cryopreservation solutions for clinical applications

**DOI:** 10.3389/fbioe.2024.1412811

**Published:** 2024-08-01

**Authors:** Yuan Tan, Mahmoud Salkhordeh, Aidan B. P. Murray, Luciana Souza-Moreira, Duncan J. Stewart, Shirley H. J. Mei

**Affiliations:** ^1^ Regenerative Medicine Program, Ottawa Hospital Research Institute, Ottawa, ON, Canada; ^2^ Faculty of Medicine, University of Ottawa, Ottawa, ON, Canada

**Keywords:** mesenchymal stem cells, cryopreservation, stability, cell therapy, final cell products, quality, off-the-shelf, potency

## Abstract

**Introduction:**

Cryopreservation is a critical process of cell products for achieving a commercial viability through wide scale adoption. By preserving cells in a lower temperature, cryopreservation enables a product to be off-the-shelf and ready for infusion. An optimized cryopreservation strategy can maintain the viability, phenotype, and potency of thawed mesenchymal stromal/stem cells (MSCs) while being regulatory compliant. We compared three clinical-ready formulations with one research cryopreservation solutions and evaluated key quality parameters of post thawed MSCs.

**Method and result:**

MSCs were cryopreserved at 3, 6, and 9 million cells/mL (M/mL) in four different cryopreservation solutions: NutriFreez (10% dimethyl sulfoxide [DMSO]), Plasmalyte A (PLA)/5% human albumin (HA)/10% DMSO (PHD10), CryoStor CS5 (5% DMSO), and CryoStor CS10 (10% DMSO). To establish post thaw viability, cells were evaluated with no dilution of DMSO (from 3 M/mL), 1:1 dilution (from 6 M/mL), or 1:2 dilution (from 9 M/mL) with PLA/5% HA, to achieve uniform concentration at 3 M/mL. Cell viability was measured at 0-, 2-, 4-, and 6-h post thaw with Trypan blue exclusion and Annexin V/PI staining. Dilution (1:2) of final cell products from 9M/mL resulted in an improvement of cell viability over 6 h but showed a trend of decreased recovery. MSCs cryopreserved in solutions with 10% DMSO displayed comparable viabilities and recoveries up to 6 h after thawing, whereas a decreasing trend was noted in cell viability and recovery with CS5. Cells from all groups exhibited surface marker characteristics of MSCs. We further evaluated cell proliferation after 6-day recovery in culture. While cells cryopreserved in NutriFreez and PHD10 presented similar cell growth post thaw, MSCs cryopreserved in CS5 and CS10 at 3 M/mL and 6M/mL showed 10-fold less proliferative capacity. No significant differences were observed between MSCs cryopreserved in NutriFreez and PHD10 in their potency to inhibit T cell proliferation and improve monocytic phagocytosis.

**Conclusion:**

MSCs can be cryopreserved up to 9 M/mL without losing notable viability and recovery, while exhibiting comparable post thaw potency with NutriFreez and PHD10. These results highlight the importance of key parameter testing for selecting the optimal cryopreservation solution for MSC-based therapy.

## Introduction

Mesenchymal stromal/stem cells (MSCs) are adult stem cells possessing unique properties that make them attractive candidates for cellular-based therapies. They can be readily isolated from both adult and neonatal tissues and expanded *in vitro*. Studies have shown that MSCs may interact with host immune host cells (Le Blanc and Ringdén, 2007; Souza-Moreira et al., 2022; Tan et al., 2023), and secrete a wide range of mediators that can stimulate repair and regeneration at the site of tissue injury (Marquez-Curtis et al., 2015; Thompson et al., 2020). Thus, there is a great interest in using MSCs to develop gene and cellular therapies for the clinic. There are currently more than 1,000 clinical trials using MSCs as cell therapy for a wide range of diseases, and more than 35% of these are using cryopreserved cells (clinicaltrial.org) (Thompson et al., 2020).

In cell therapy, cryopreserved products have captured significant interest by providing off-the-shelf flexibility, which can be advantageous over culture of fresh cells. A more extensive quality control strategy could be incorporated into a standardizable production process, which allows for a consistent supply tailored for timely therapeutic delivery (Bahsoun et al., 2019; Woods et al., 2016; Yuan et al., 2016). Cryopreserved allogeneic MSCs have been used widely to date (Thompson et al., 2020); however, without careful optimization, the process of cryopreservation can reduce cell viability, recovery and potency after thaw (Wick et al., 2021). Parameters such as the choice of cryopreservation solutions and cell processing procedures are essential to ensure consistent product efficacy and stability.

One of many important considerations during cryopreservation protocol development is the clinical utility and safety of cryopreservation agents (CPA) that are appropriate to use in patients. Although there is a continuing effort to search for alternative CPA, dimethyl sulfoxide (DMSO), a small molecule that acts as a permeating CPA (Ekpo et al., 2022), is currently one of the most commonly used cryoprotectants in cryopreserved MSC products (Ock and Rho, 2011; Verheijen et al., 2019). Typically, if cells undergo cryopreservation without any CPA, cell damage can occur through two widely accepted mechanisms of action: 1) formation of intracellular ice crystals that weaken cell membrane integrity upon thaw, and 2) as water organizes itself into crystal lattices, solute concentrations increase in unfrozen parts of the solution, causing osmotic imbalances (Acker, 2005; Whaley et al., 2021). DMSO minimizes these cryo-injuries through its strong hydrogen bonding interactions with water, disrupting ice crystallization formation and preventing the dangerous intra- and extra-cellular increases in solute concentration (Acker, 2005; Shivakumar et al., 2016). If used at extremely high concentrations (i.e., 40%), DMSO can interrupt cell membrane stability (Gurtovenko and Anwar, 2007) and has been reported to cause adverse events in patients (Gurtovenko and Anwar, 2007; Alessandrino et al., 1999; Rowley et al., 1999). Clinically used MSC products are commonly cryopreserved in a solution with 5%–10% of DMSO, which is also the concentration range used in preserving blood and hematopoietic transplant products without significant safety issues (Barnes and Loutit, 1955; Fleming and Hubel, 2006; Berz et al., 2007). Diluting DMSO concentration prior to product infusion can further reduce safety concerns but may require MSCs to be cryopreserved at a high cell concentration, which might compromise cell number recovery and viability after thaw (Fry et al., 2015).

To date, many clinical trials are testing MSCs as a potential therapy due to their putative immunomodulatory properties. While some studies have reported that low cell viability post-cryopreservation and thawing impaired MSC functionalities (Galipeau, 2013; Pollock et al., 2015; Giri and Galipeau, 2020), others demonstrated that there is no significant difference in MSCs products that had been used after fresh culture/harvest *versus* cryopreservation and thaw, with both exhibiting similar immunoregulatory functions (Tan et al., 2019; Horiuchi et al., 2021). Herein, our study compares four different cryopreservation regimens: one in-house formulation (a common formulation for a MSC final product) (Petrenko et al., 2019) and three proprietary pre-formulated cryopreservation solutions. We assess the effect of each of these cryopreservation solutions on cell viability, recovery, phenotype, and immunomodulatory functions of MSCs post thaw.

## Materials and methods

### MSCs culture

Bone marrow from one donor was purchased through a commercial supplier (Lonza, Walkersville), while two other bone marrow aspirates were obtained from healthy volunteer donors through The Ottawa Hospital, Ottawa, Canada, with informed consent and ethical approval granted by The Ottawa Health Science Network Ethics Board (REB ID: 20120929-01H). MSCs were isolated and cultured in Nutristem XF complete media (Sartorius, United States) and cryopreserved in four different cryopreservation solutions: 1) NutriFreez (NutriFreez D10, containing 10% DMSO) (Sartorius, United States); 2) PHD10 (plasmalyte-A [PLA; pH 7.4, Baxter] supplemented with 5% human albumin [HA; Alburex 25, CSL behring] and 10% DMSO [Thermofisher, United States]); 3) Cryostor CS5 (containing 5% DMSO; Biolife solutions); 4) Cryostor CS10 (containing 10% DMSO; Biolife solutions). For each cryopreservation solution, cells were frozen at concentrations of 3 million cells/mL (M/mL), 6 M/mL, and 9 M/mL. All experiments used cryopreserved MSCs at passage 4. The capacity of these MSCs to differentiate into adipocytes, osteocytes, and chondrocytes was previously demonstrated (Tan et al., 2019).

### Thaw and dilution

After storage at liquid nitrogen (>1 week), each vial of MSCs was thawed by placing vials into a 37°C water bath for 2 min. Cells cryopreserved at a 3 M/mL concentration were thawed, and the cell count, and viability measurement were performed without dilution. After thawing, cells cryopreserved at a 6 M/mL concentration were followed by equal volume (1:1) dilution with PLA/5%HA, while cells at 9 M/mL were diluted 1:2 (to reach final concentration of 3 M/mL) before testing. MSCs were left at room temperature (up to 6 h) for the time-course study. MSC recovery was calculated by dividing cell number taken at every time point by the concentration cells were cryopreserved at.

### Viability assessment

Cell viability was assessed using the Trypan blue exclusion method, and apoptosis was measured by Annexin V (AV) and propidium iodide (PI) staining, followed by flow cytometry analysis. Viable cell recoveries were calculated by dividing the total number of live cells counted by the number of cells originally cryopreserved in a vial. For flow cytometry analysis, cells were stained with AV and PI for 15 min at room temperature according to manufacturer’s instructions before putting samples through the flow cytometer (Attune Acoustic Focusing cytometer, Thermofisher). The data analysis was conducted using FlowJo X software (FlowJo, LLC).

### MSCs surface marker analysis

Immune-phenotype of MSCs was analyzed for the surface marker expression of PE conjugated CD73, CD90, and CD105, CD14, CD19, CD34, CD45, and HLA-DR (BD Pharmingen) expression with matching isotype controls. After thawing, MSCs were washed and resuspended in cold PBS supplemented with 3% fetal bovine serum (FBS, Life Technologies) and stained according to antibodies’ manufacturer instructions. After washing, MSC surface markers were detected by a flow cytometer and analyzed by FlowJo 10.0 software (FlowJo, LLC).

### Post thaw subculture

Cryopreserved cells were thawed and seeded at a density of 1,000 cells/cm^2^ to assess post thaw cell recovery and ability to be culture expanded. To assess cell proliferative potential after cryopreservation, cells were in culture with Nutristem XF complete media for 6 days. On Day 6, cells were harvested using TrypLE (Gibco, Thermofisher) and counted using Trypan blue exclusion method. Fold of increase was calculated by dividing the final cell number by the initial seeding number.

### Potency assay

The inhibition of T-cell proliferation assay used peripheral blood mononuclear cells (PBMCs) stained with carboxyfluorescein succinimidyl ester (CFSE, Fisher Scientific) and activated with Human T-Activator CD3/CD28 (Dynabeads, Gibco). Cryopreserved MSCs were in culture for 24 h prior to being co-cultured with the activated PBMCs for 5 days, followed by assessment by flow cytometer.

In the phagocytosis assay, MSCs were seeded for 24 h prior to being co-cultured with PBMCs. PBMCs used were first pre-treated with lipopolysaccharides (LPS; 100 ng/mL), followed by co-culture with the MSC groups for 24 h. After co-culture, PBMCs were harvested, incubated with green fluorescent-tagged *E. coli* Bioparticles (pHrodo; Invitrogen) and then stained with mouse anti-human CD14 conjugated with BV421 antibody (BD Biosciences) for dye-based detection of phagocytosis by Attune Acoustic Focusing cytometer (Invitrogen).

### Statistical analysis

Statistical analysis was performed using GraphPad Prism V10.0 software (GraphPad Software, San Diego). Numerical data are presented as mean ± SEM unless otherwise stated. Multiple groups were analyzed by one-way ANOVA followed by Dunnett’s multiple comparisons test unless otherwise stated. Statistical significance was set at *p* < 0.05.

## Results

### Comparison of trypan blue viability and recovery of MSCs cryopreserved in four cryopreservation solutions

**FIGURE 1:**
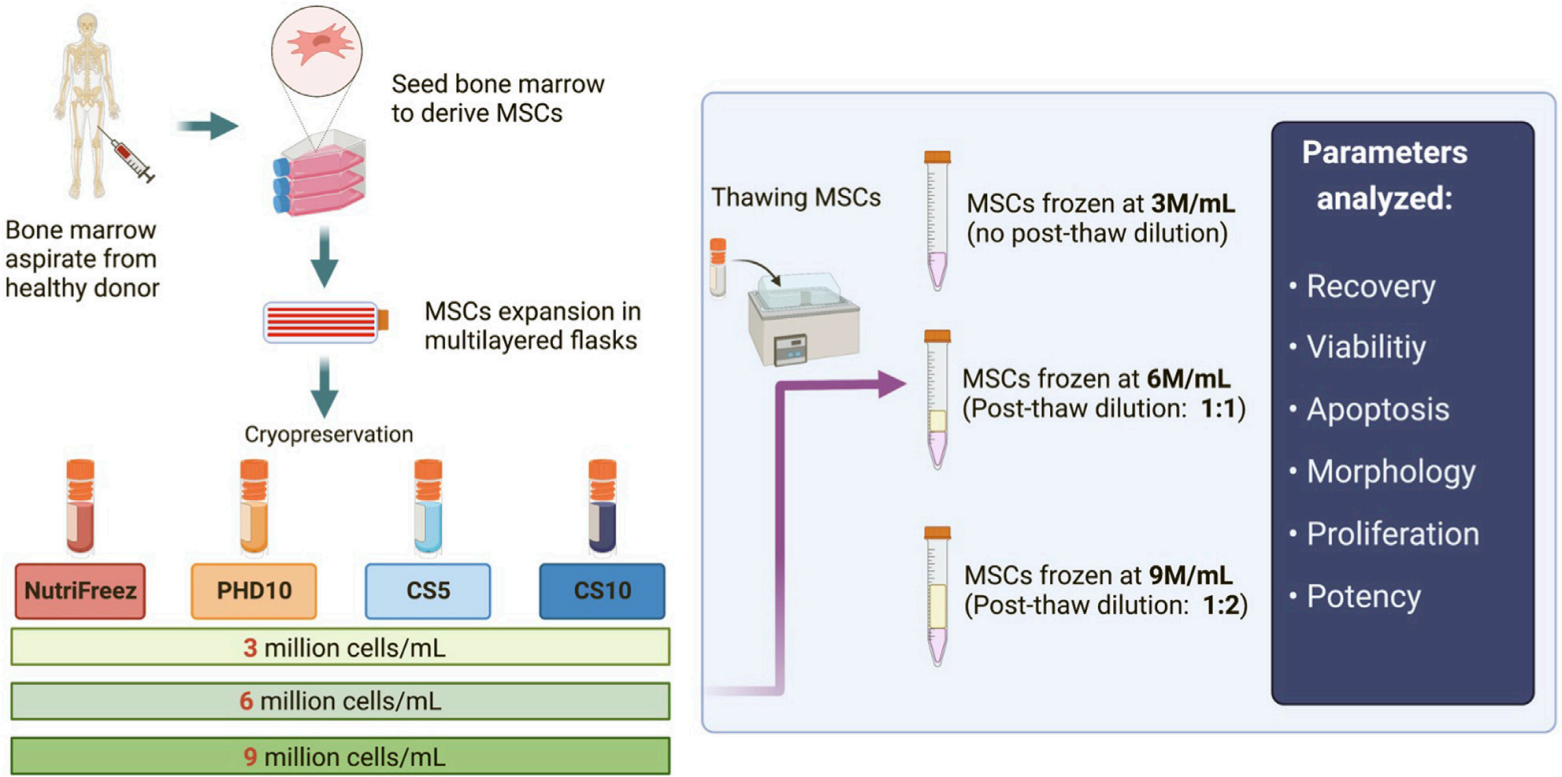
Schematic of the experimental design for MSC isolation, culture expansion, and cryopreservation. Bone marrow aspirates were first obtained from healthy donors, and MSCs were derived, expanded, and cryopreserved in four different cryopreservation solutions: NutriFreez, PHD10 (Plasmalyte-A, 5% human albumin, 10% DMSO), Cryostor CS5, and Cryostor CS10. For each cryopreservation solution, cells were frozen at concentrations of 3 million cells/mL (M/mL), 6 M/mL, or 9 M/mL. Cryopreserved MSCs were thawed, with or without dilution (pending on freezing cell concentrations), for downstream analysis.

To determine the effects of different cryopreservation solutions and cell concentrations on MSC viability and recovery, bone marrow-derived MSCs were prepared as described in Figure 1. Four different cryopreservation solutions were tested, three of which contained 10% DMSO (NutriFreez, PHD10, and CS10) and one with 5% DMSO (CS5). The final targeted cell concentration was set at 3 million cells per mL (M/mL). For cells cryopreserved at higher concentrations (6M/mL or 9M/mL), post thaw dilution with equal or double volume of PLA/5%HA was used to reach the final cell concentration at 3 M/mL.

**FIGURE 2:**
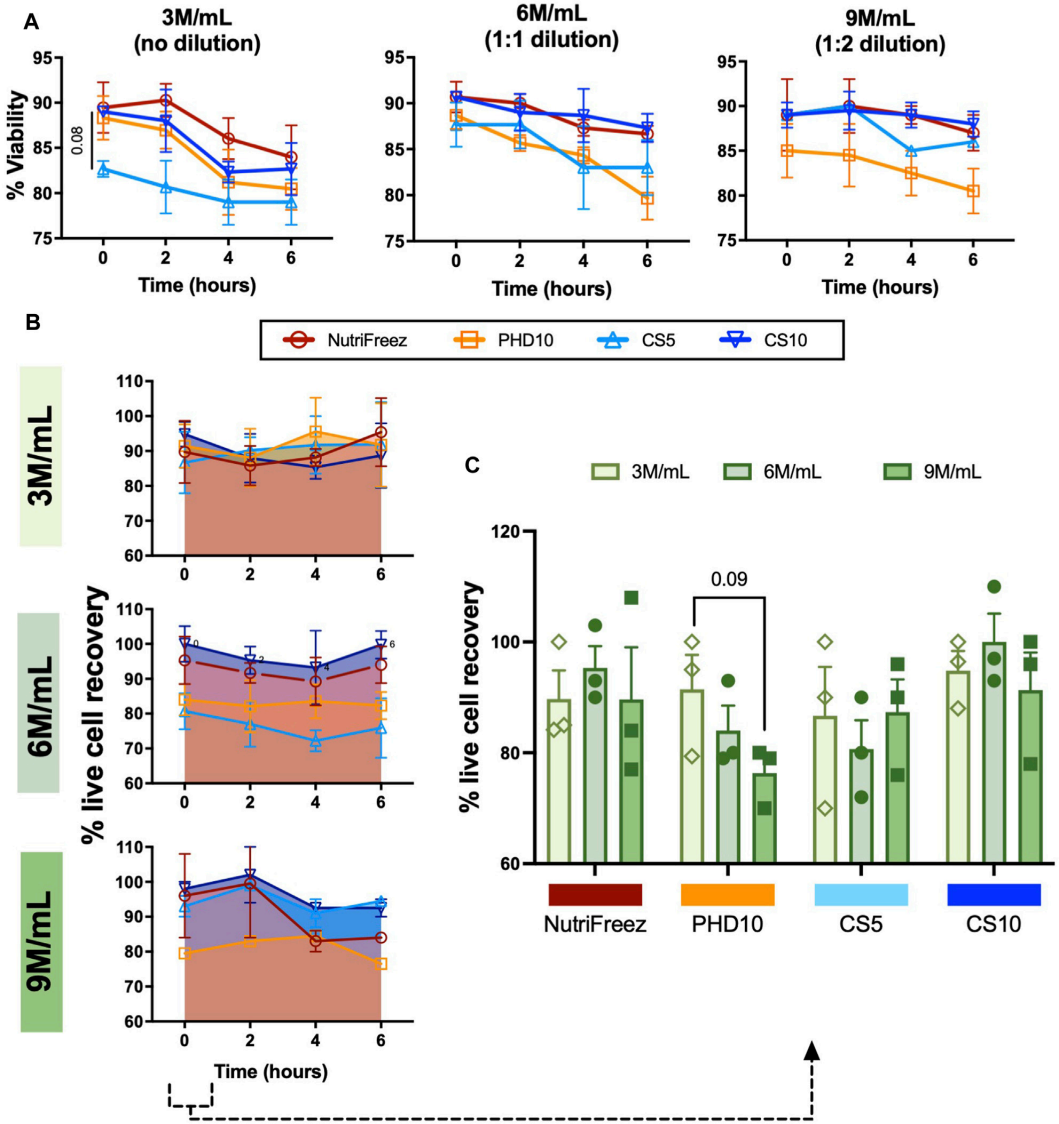
Assessment and comparison of MSC viability and recovery post thaw over 6 h. Trypan blue exclusion was used to assess **(A)** post thaw viabilities and **(B)** viable cell recoveries, and **(C)** viable cell recoveries at 0h post thaw. Aliquot of cells were collected and used for measurements at 0, 2, 4, and 6 h n = 3 independent experiments with MSCs from one donor, data graphed as mean ± SEM. Group comparisons were analyzed by one-way ANOVA with Dunnett’s *post hoc* test. For A, *p* = 0.08 comparing CS5 vs. Nutrifreez D10.

Immediately post thaw, MSCs frozen at 3M/mL (no dilution) in NutriFreez, PHD10 and CS10 displayed similar percentage viabilities at 89.3% ± 4.8%, 88.1% ± 4.2%, and 89.3% ± 0.2%, respectively, whereas cells cryopreserved with 5% DMSO (CS5) showed a modest lowered viability (82.7% ± 1.5%), compared to the other three solutions (0-h post thaw, Figure 2A). Furthermore, we tested MSC cryopreservation at different cell concentrations and showed that MSCs cryopreserved in all tested solutions showed >80% viability immediately post thaw (Figure 2A). To assess in-use stability, thawed MSCs were left at room temperature over 6 h, with samples taken at every 2 h to assess parameters demonstrative of cell product stability. There was an average decrease of 5.5%, 7.9%, 3.7%, and 6.3% in viability of cells cryopreserved at 3M/mL in NutriFreez, PHD10, CS5, and CS10, respectively (Figure 2A) over 6 h at room temperature. There was a slight and non-significant reduction in cell viability across all tested cryopreservation solutions and cryopreserved cell concentrations over 6 h, which was more evident in the 3M/mL group compared to the 6 and 9M/mL groups (Figure 2A).

Immediately post thaw, live cell recovery of 90%, 93%, 85%, and 91% were recorded for cells cryopreserved at 3M/mL for NutriFreez, PHD10, CS5, and CS10, respectively (Figure 2B). Although there was some variability, cells cryopreserved at higher concentrations (6M/mL and 9M/mL) did not show significant loss in cell recovery immediately post thaw (Figure 2C). In addition, there was no significant decline of cell recovery post thaw over 6h amongst the four cryopreservation solutions and three concentrations tested (Figure 2B). However, a decreasing trend in cell recovery in PHD10 was noted as cell concentration increased to 9M/mL immediately post -thaw (0h) (Figure 2C). Overall, we revealed that MSCs can be cryopreserved up to 9 M/mL with good cell viability and recovery, and cryopreservation reagents containing 10% DMSO displayed superior cryopreservation outcomes in viability and cell recovery.

### Assessment of apoptosis levels in cryopreserved MSCs post thaw

**FIGURE 3:**
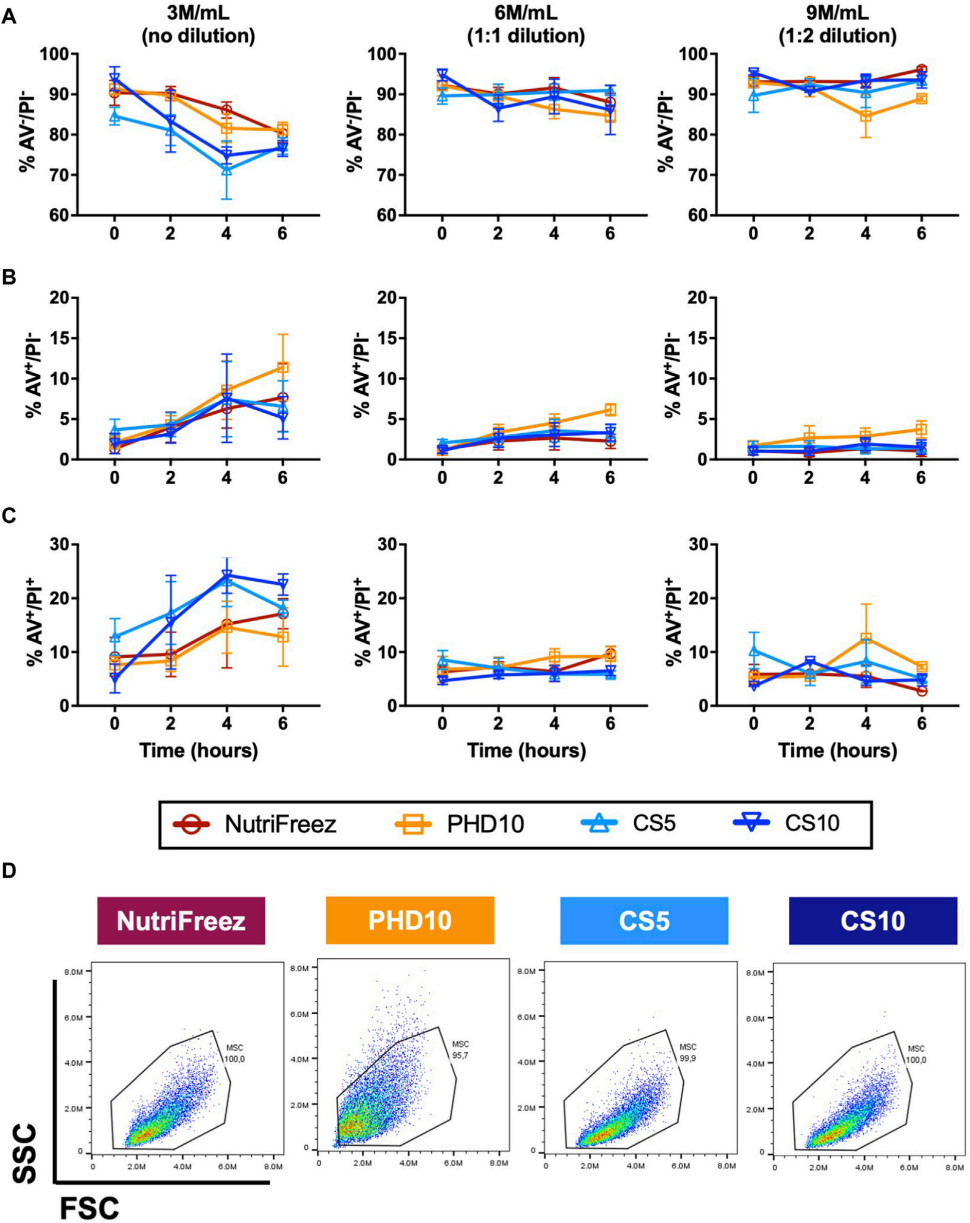
Assessment and comparison of apoptosis levels in cryopreserved MSC over 6 h post thaw. Annexin V (AV) and PI staining was performed on the MSCs that had been cryopreserved in NutriFreez, PH10, CS5, and CS10. Flow cytometry analysis was carried out to assess levels of cellular apoptosis at 0, 2, 4, and 6 h. Data represents **(A)** live cells from AV-/PI- population, **(B)** early apoptotic cells from AV+/PI- population, and **(C)** dead cells from AV+/PI + population. **(D)** Representative forward scatter (FSC) and side scatter (SSC) from each cryopreservation conditions. n = 3 independent experiments with MSCs from one donor, data plotted as mean ± SEM.

Flow cytometry analysis of AV/PI staining was performed to assess levels of MSC apoptosis in all groups. MSCs cryopreserved at 3M/mL at lower concentrations of DMSO (5% DMSO, CS5) had slightly lower cell viability at 0 h compared to MSCs cryopreserved with 10% DMSO (NutriFreez, PHD10, and CS10). Immediately post thaw, the percentage of MSCs cryopreserved at 3M/mL with AV-/PI- were at 90% ± 5.3% for NutriFreez, 91% ± 2.3% for PHD10, 85% ± 3.7% for CS5, and 94% ± 5.0% for CS10, respectively (Figure 3A). Over the course of 6 h, non-apoptotic/non-necrotic (AV-/PI-) cells at 3M/mL NutriFreez showed decline in AV-/PI- population from 90% ± 5.3% immediately post-thaw to 80% ± 3.8% at 6 h, 3M/mL PHD10 from 90% ± 2.3% to 81% ± 3.3%, 3M/mL CS5 from 84% ± 3.8% to 77% ± 3.6%, and 3M/mL CS10 from 94% ± 5.1% to 76% ± 3.4%. Over 6 h, cell viability in all groups showed decreases in AV-/PI- population and increases in apoptotic cells (AV+/PI- cells) (Figure 3B) and necrotic cells (AV+/PI+) (Figure 3C), with none reaching statistical significance. In MSCs cryopreserved at 6 or 9 M/mL, which were diluted post thaw to reduce % DMSO levels to 5% or 3.3% in the final formulation, we observed a small decline in AV-/PI- viable cells over time. Viability of MSCs cryopreserved at 6M/mL PHD10 was at 92% ± 4.3% at 0 h, which declined to 84% ± 2.7% by 6 h, while cell viability post cryopreservation at 9M/mL ranged from 92% ± 0.6% at 0 h, to 89% ± 0.9% at 6 h (Figures 3A–C). Morphological characteristics were also examined at 0-h post thaw via flow cytometry (size by FSC and granularity by SSC). No differences in cell size and granularity were observed in cells cryopreserved in NutriFreez, CS5, and CS10 while MSCs cryopreserved in PHD10 showed a slight increase in granularity, based on side scatter assessment via flow cytometry, in all time points evaluated (Figure 3D; Supplementary Figure S1). Therefore, MSCs cryopreserved in four tested cryopreservation solutions demonstrated comparable cell viability measured via AV/PI, with all conditions maintaining >75% non-apoptotic cells even at 6 h post thaw. Dilution of the cell product post thaw (i.e., cryopreserved MSCs at 10% DMSO, but reduced DMSO by post thaw dilution) rendered a better in-use stability with less cellular apoptosis over 6 h.

### Characterization and subculture of MSCs in different cryopreservation solutions

**FIGURE 4:**
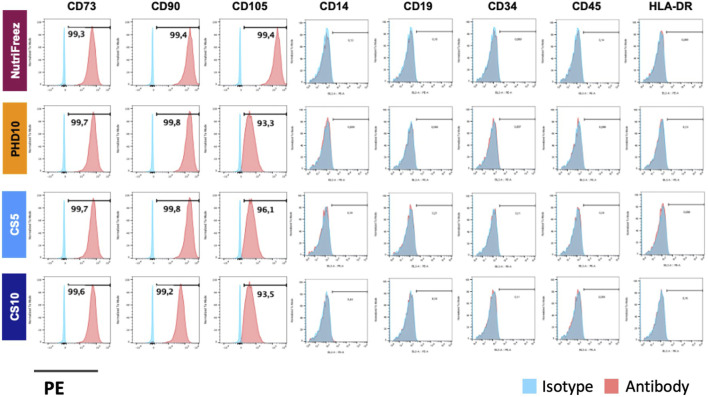
Surface marker characterization of cryopreserved MSCs. Representative flow cytometric plots indicate positive markers (CD73, CD90, CD105) and negative markers (CD14, CD19, CD34, CD45, and HLA-DR) for MSC characterization profile. n = 3 independent experiments with MSCs from one donor.

**FIGURE 5:**
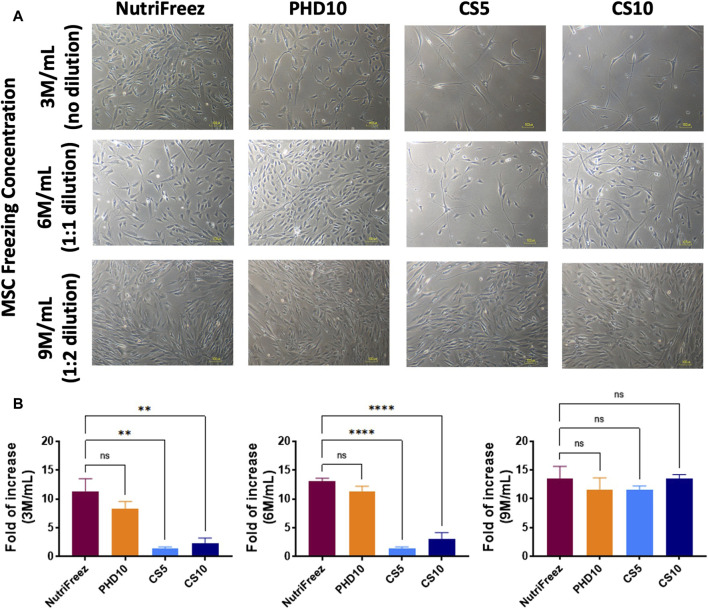
Post thaw expansion of cryopreserved MSCs. Post thaw, MSCs were seeded and cultured for 6 days to examine **(A)** cell morphology, and **(B)** proliferation potential (fold of cell number increases, calculated by dividing the number of cells harvested at day 6 to the number of cells used at initial seeding). Scale bar = 100 μm n = 3 independent experiments with MSCs from one donor, data plotted as mean ± SEM. Group comparisons were analyzed by one-way ANOVA with Dunnett’s *post hoc* test, *P < 0.05, **P < 0.01, and ****P < 0.0001.

Surface marker profile was assessed immediately post thaw and showed consistent expression of CD73, CD90, and CD105 (>90%); and negative expression of CD14, CD19, CD34, CD45, and HLA-DR (<2%), confirming the MSC identity in all groups (Figure 4). To check whether the recovered viable cells maintained proliferative potentials, we seeded the thawed MSCs immediately post thaw and cultured cells for 6 days. Comparable cell growth was observed with no abnormal cell morphology in MSCs cryopreserved in NutriFreez or PHD10 across all concentrations (3, 6, or 9 M/mL). However, there was notable impairment in cell attachment with elongated morphology seen for cells cryopreserved in CS5 and CS10 (Figure 5A). Significantly lower folds of expansion were also noted for MSCs that had been cryopreserved in both CS5 and CS10, compared to NutriFreez at both 3M/mL and 6M/mL (Figure 5B). In MSCs cryopreserved at 9M/mL, the levels of cell expansion were comparable across all groups. In summary, our data showed that MSCs cryopreserved in NutriFreez and PHD10 demonstrated comparable surface marker characterization and proliferation potentials regardless of cell concentration.

### Comparison of MSC immune potency after cryopreservation

**FIGURE 6:**
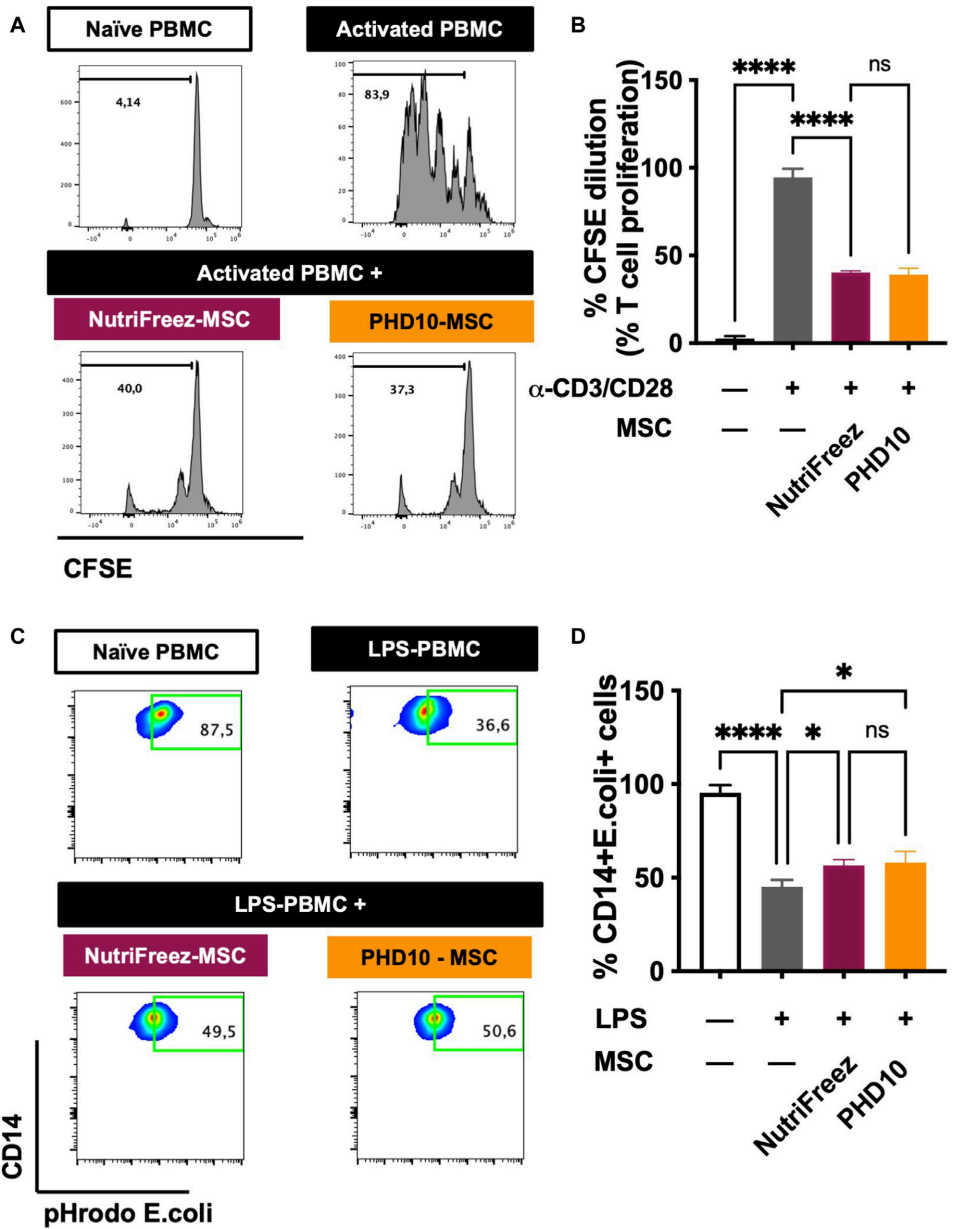
Effect of MSCs on the inhibition of T-cell proliferation and improvement of monocytic phagocytic capacity post LPS injury. **(A)** Representative flow cytometric plots of CFSE dilution of naïve PBMCs, CD3/CD28 activated PMBCs without and with MSCs co-culture (previously cryopreserved in NutriFreez or PHD10), showing the ability of MSC to inhibit T-cell proliferation. **(B)** Quantification and summary data is plotted as a bar graph, compared to negative control of naïve PBMCs and activated PBMCs. **(C)** Representative flow cytometric plots of naïve PBMCs, LPS-treated PBMCs without and with MSCs (previously cryopreserved in NutriFreez or PHD10) demonstrating the PBMC’s ability to phagocytose bacteria as indicated by the percentage of CD14^+^ cells positive for green, fluorescent signal. **(D)** Quantification and summary data is plotted as a bar graph, compared to negative control of naïve PBMCs and activated PBMCs. n = 3 independent experiments using three different donors derived MSCs, data represent mean ± SEM. Group comparisons were analyzed by one-way ANOVA with Tukey’s *post hoc* test, *P < 0.05 while ns = non-.significant.

Immunomodulatory effects of MSCs have been documented showing MSCs can suppress the proliferation of activated T cells and enhance the phagocytic abilities of LPS injured monocytes (Meisel et al., 2004; Mei et al., 2012; Tan et al., 2019). Having established that comparable good viability and post thaw proliferation recovery obtained by NutriFreez solution and PHD10 cryopreserved at 6M/mL, these two cryopreservation solutions were subsequently selected for potency analysis. After 5 days of co-culture with activated PBMCs, MSCs that had been cryopreserved in NutriFreez and PHD10 displayed comparable inhibitory capacity on T cell proliferation (Figures 6A, B). We further tested the ability of MSCs to rescue monocyte phagocytosis by co-culturing MSCs with LPS-injured PBMCs. While naïve monocytes demonstrated high phagocytic activities of pHrodo *E. coli* pseudoparticle, LPS-injured CD14^+^ monocytes had impaired phagocytosis. After co-culturing with MSCs (either from NutriFreez or PHD10), monocytes’ phagocytic capacity was significantly improved (*p* < 0.05 vs. LPS-injured monocytes without MSCs, Figures 6C, D). Importantly, there was no significant difference between MSCs cryopreserved in NutriFreez or PHD10 regarding their abilities to rescue monocyte phagocytosis.

## Discussion

In the current study, we provided a comprehensive assessment of the effect of different cryopreservation solutions and cell concentrations on cell viability, recovery, characterization, immunomodulatory potency, and in-use stability of MSCs. We demonstrated that cryopreserved MSCs in three commercially available cryopreservation solutions (NutriFreez, CS5, and CS10) and in-house defined media (PHD10), with up to 9 M/mL cryopreservation concentration, showed no significant loss in cell viability, recovery, or surface marker phenotype post thaw. Cells cryopreserved in CS5 (lower DMSO concentration) showed a trend towards decreasing cell viability immediately post thaw. More importantly, in addition to improved levels of the quality parameters mentioned above, cells cryopreserved in NutriFreez and PHD10 demonstrated comparable potency in modulatory potential, which included the ability to inhibit T cell proliferation and rescue monocyte phagocytosis after LPS injury.

Although there was a positive correlation between increasing DMSO concentrations and preserving cell viability during the cryopreservation process, prolonged exposure of cells to high levels of DMSO (over 40%) has been shown to damage cells upon thawing and negatively impact final cell product viability (Rubin, 1997; Alessandrino et al., 1999; Fry et al., 2015). As DMSO is hyperosmotic, osmotic fluctuation after thawing of cryopreserved cells can lead to excessive cell expansion and decreased cell viability. This has been implicated in causing impairment of long-term engraftment in hematopoietic stem cell transplants (Rowley et al., 2003; Shu et al., 2014). Currently, cryopreservation of MSCs is commonly performed using DMSO between the range of 2%–10% for research-grade and clinical products (Erol et al., 2021). While lower concentration of DMSO (less than 10%) may cause less toxicity to the cells, in our study this resulted in a decrease in cell viability compared to cryopreservation solutions with 10% DMSO. Similarly, Ginis *et al.* showed while no significant difference in post thaw viabilities between 2% and 10% DMSO after a shorter storage period (less than 1 month), a significantly higher percentage of dead cells was seen in cells cryopreserved in 2% DMSO after 5-month storage (Ginis et al., 2012).

Addition of a dilution step after cell product thawing could minimize exposure of higher concentrations of DMSO (i.e., 10% or higher) on thawed cells. When considering practical aspects of any cryopreserved cell product, the ability to maintain post thawed cell viability over an extended period can increase the flexibility in the drug administration window at the bedside. In all conditions tested here, a decrease in post thaw viability over time was observed; however, cell viability was better maintained after dilution of DMSO concentrations to below 10% (to 5 or 3.3% in final formulation). This is consistent with a study by Fry and colleagues, in which they reported that reducing DMSO concentration (by diluting cryopreserved core blood mononuclear cells) improved recovery of viability in final cell product over the course of 48 h post thaw, compared to undiluted cells. They showed that washed cell product exhibited the highest preservation of viability (over 80% viable cells) up to 24 h post thaw; however, the washing step also resulted in significant decreases in the number of recovered cells (Fry et al., 2015). In a published clinical trial that had implemented a wash step post product thaw, they reported wide range in recovery viabilities of the final cell products (from 30% to 80%) (Matthay et al., 2019), which may potentially affect the clinical outcomes of the trial.

It has been reported that compared to cultured cells, some studies showed that the thawed MSCs could trigger an innate immune response and activate the complement cascade (Moll et al., 2014; Moll et al., 2016; Moll et al., 2019; Cottle et al., 2022). However, we and others have also demonstrated, using *in vitro* assays and *in vivo* models, cryopreserved/thawed MSCs isolated from bone marrow can exert comparable immune functionalities as that of fresh cultured cells (Cruz et al., 2015; Tan et al., 2019). Differences in the origins of tissue source, age of the tissue donor, methods of MSC isolation and culture, passage number of the cells and cryopreservation processes may contribute to the different observations seen in these studies.

In this study, we have included cryopreservation formulations that are clinically relevant to MSCs final cell products. Other than NutriFreez, which is not suitable for direct injection into patients, both CS5 and CS10 have been previously demonstrated to protect against cell damage, and maintain a high cell viability and recovery for patient use (Clarke and Lawrence, 2019; Linkova et al., 2022). Additionally, CS10 is used as an excipient component in food and drug agency-approved CAR T-cell therapies (Abramson et al., 2020; Shah et al., 2021; Neelapu et al., 2022). Another relevant formulation included in this study is comprised of PLA, HA, and DMSO, which is commonly used in clinical settings and routinely used in blood transfusion (Hare et al., 2009; Weiss et al., 2013).

We also found that cells cryopreserved in CS5 and CS10 exhibited lower expansion capacities after 6 days of culture, particularly when cryopreserved at 3M and 6M/mL. While other studies have demonstrated that CS5-cryopreserved MSCs had lower metabolic activity (via XTT assay based on reduction of tetrazolium salt to formazan dye) at up to 72 h post-seeding compared to seeding with fresh culture MSCs, no statistically significant difference was found between both groups (Gramlich et al., 2016). In contrast, a study performed by Ginis et al. found that seeding MSCs that had been cryopreserved in CS5 and CS10 showed comparable or even increased proliferation rates, compared to cells that had not been cryopreserved. They also reported that cells cryopreserved in CS5 or CS10 exhibited no differences to surface markers consistent with an MSC cell type, thereby eliminating the possibility of different cell types expressing different proliferation rates (Ginis et al., 2012). Of note, in our study, the MSCs that had been cryopreserved at 9M/mL in CS5 and CS10 cells and diluted 1:2 prior to seeding did not show significant loss in cell proliferation rates.

For MSC therapies that are designed to treat immune disorders, the ability to retain immunomodulatory potency after a cryopreservation and thaw process can ensure that the potency of MSCs has been preserved. We have previously reported that MSCs cryopreserved in NutriFreez retained their capabilities of inhibiting T-cell proliferation and improving monocyte phagocytosis after LPS injury at a similar extent to the cultured cells (Tan et al., 2019). In this study, having established that thawed cells in NutriFreez and PHD10 shown comparable viability and post thaw recovery, we next examined whether they also preserve the functionality of MSCs. We found that no differences were observed in T cell inhibition or rescue of monocyte phagocytosis in cells cryopreserved in 6M/mL, compared to cells that were cryopreserved with PHD10. The equivalent ability of PHD10 (widely used in many MSC clinical trials) to maintain immunomodulatory potency as NutriFreez is shown. As other cryopreservation solutions have limitations either in clinical application (NutriFreez) and impaired cell growth post thaw (CS5 and CS10), our data suggested that PHD10 may be one of the most suitable cryopreservation solutions for clinical application of MSC therapy. Mounting evidence from Phase I and compassionate use clinical trials has demonstrated safety and tolerability in patients who had received infusions of MSCs suspended in PLA solutions with or without HA (Marquez-Curtis et al., 2015; Kamen et al., 2018). Our results here further demonstrated that the use of PHD10 allows the maintenance of MSC potency post thaw after cryopreservation and is clinically applicable without compromising on efficacy of MSC therapies.

## Conclusion

In summary, the current study evaluated three clinically relevant cryopreservation formulations (CS5, CS10, and PHD10) and compared these to a well-performed, research grade cryopreservation solution NutriFreez. Our data demonstrated that MSCs can be successfully cryopreserved in all formulations tested across different cell freezing concentration, while the introduction of a post thaw dilution step can further improve in-use stability. Optimized cryopreservation and post thaw preparation of the final cell product retained MSCs immune properties post thaw. Overall, these results provide insights to inform the choice of cryopreservation solutions and cell concentration for cell therapy products for use in clinical trials, while highlighting that the key quality parameters need to be examined to fully define cryopreservation and thaw effects on clinical ready MSC products for therapeutic evaluation.

## Data Availability

The raw data supporting the conclusions of this article will be made available by the authors, without undue reservation.
