# Supplementary material for: Key quality parameter comparison of mesenchymal stem cell product cryopreserved in different cryopreservation solutions for clinical applications
Source: Front Bioeng Biotechnol. 2024 Aug 1;12:1412811. doi: 10.3389/fbioe.2024.1412811 (PMC11324487; doi:10.3389/fbioe.2024.1412811)
Supplement: Supplementary file 1 [file DataSheet1.docx]

Supplementary Material

Key Quality Parameter Comparison of Mesenchymal Stem Cell Product Cryopreserved in Different Cryopreservation Solutions for Clinical Applications

Yuan Tan^1, 2^†, Mahmoud Salkhordeh^1^†, Aidan B.P. Murray^1^, Luciana Souza-Moreira^1^, Duncan J. Stewart^1,2^, Shirley H.J. Mei^1*^

*** Correspondence:**Shirley H.J. Mei
smei@ohri.ca

# Supplementary Figures and Tables

## Supplementary Figures


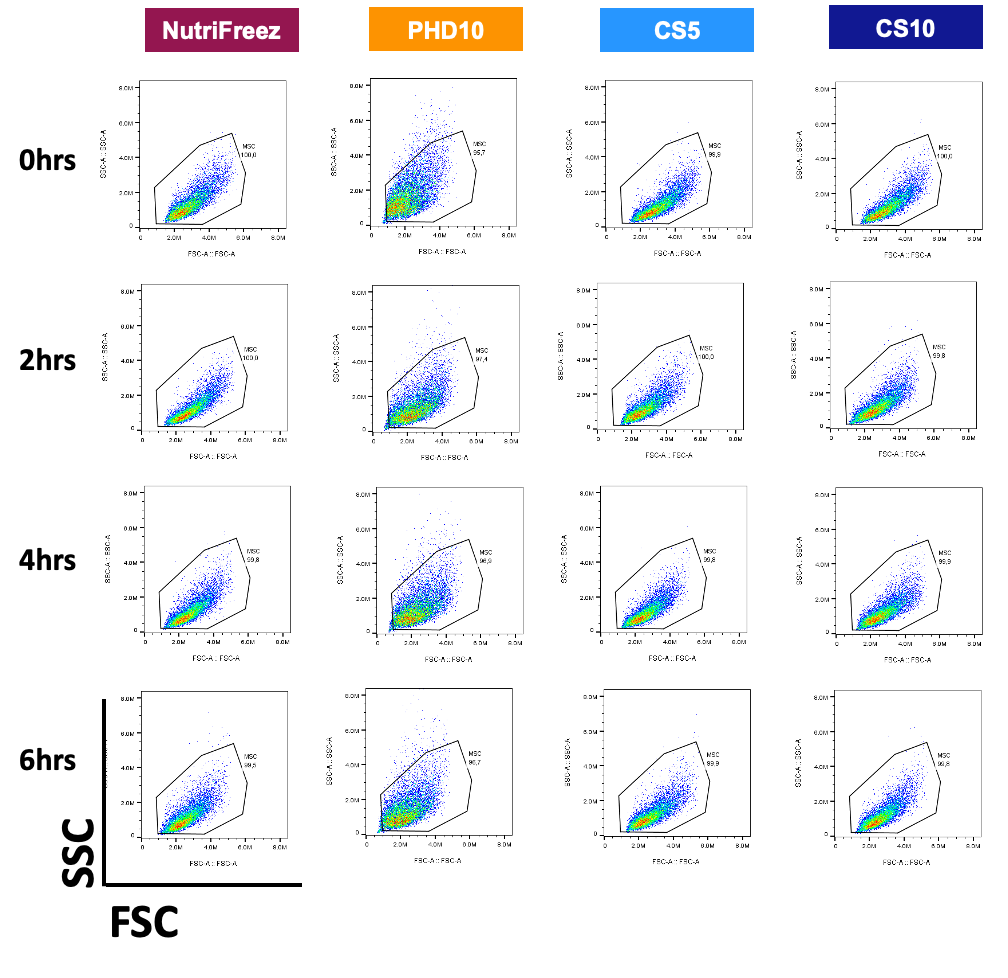


**Supplementary Figure 1.** cell size and granularity of MSCs cryopreserved in four cryopreservation solution 6-hour post thaw.
